# Understanding How Health Providers Identify Women with Postpartum Hemorrhage: A Qualitative Study

**DOI:** 10.1055/s-0041-1733997

**Published:** 2021-10-20

**Authors:** Silvana Ferreira Bento, Anderson Borovac-Pinheiro, Erika Zambrano Tanaka, Carla Silveira, Rodolfo Carvalho Pacagnella

**Affiliations:** 1 Universidade Estadual de Campinas, Campinas, SP, Brazil

**Keywords:** postpartum hemorrhage, diagnosis, clinical judgment, qualitative research, health professionals, hemorragia pós-parto, diagnóstico, julgamento clínico, pesquisa qualitativa, profissionais de saúde

## Abstract

**Objective** To identify how health providers recognize postpartum hemorrhage early and the difficulties involved in it.

**Methods** An exploratory, descriptive study using a qualitative approach through a semi-structured interview technique. In total, 27 health professionals (nursing technicians, nurses, medical residents in Gynecology and Obstetrics, hired medical doctors, and medicine professors) working in a tertiary-level hospital of reference in women's health care in the State of São Paulo, Brazil, participated in the study through an invitation. After they accepted the invitation, they signed the free and informed consent form. All interviews were recorded and transcribed, and a thematic analysis was conducted. We found three analysis categories: a) perception of the severity: “there is something wrong with the women”; b) difficulties in the early diagnosis of postpartum hemorrhage; and c) the process to improve obstetrical care.

**Results** Caregivers believe teamwork and communication should be improved. Besides the visual estimation of blood loss, the nursing team is attentive to behavioral symptoms like irritability, while the medical staff follow protocols and look for objective signs, such as altered vital signs.

**Conclusion** Besides the objective evaluations, the subjective perceptions of the providers are involved in the clinical judgement regarding the diagnosis of postpartum hemorrhage, and this should be included in a broader diagnosis strategy.

## Resumo

**Objetivo** Identificar como os profissionais de saúde reconhecem precocemente os casos de hemorragia pós-parto e as suas dificuldades.

**Métodos** Realizou-se um estudo exploratório, descritivo, com uma abordagem qualitativa por meio da técnica de entrevista semiestruturada. Por meio de um convite, participaram do estudo 27 profissionais saúde (técnicas de enfermagem, enfermeiras, residentes de Ginecologia e Obstetrícia, e médicos contratados e docentes) que trabalhavam em um hospital de nível terciário de referência no atendimento à saúde da mulher no estado de São Paulo. Depois que os participantes aceitaram o convite, eles assinaram o termo de consentimento livre e esclarecido. Todas as entrevistas foram gravadas, transcritas, e realizou-se uma análise temática. Identificaram-se três categorias de análise: a) percepção da gravidade: “há algo de errado com as mulheres”; b) dificuldades no diagnóstico precoce da hemorragia pós-parto; e c) o processo para melhorar a atenção em obstetrícia.

**Resultados** Os profissionais de saúde acreditavam que o trabalho em equipe e a comunicação deviam ser aperfeiçoados. Além da estimativa visual da perda de sangue, a equipe de enfermagem estava atenta a sintomas comportamentais como irritabilidade, ao passo que o pessoal médico seguia protocolos e procurava sinais objetivos, como sinais vitais alterados.

**Conclusão** Além das avaliações objetivas, as percepções subjetivas dos provedores estão envolvidas no julgamento clínico do diagnóstico de hemorragia pós-parto, e isto deve ser incluído em uma estratégia de diagnóstico mais ampla.

## Introduction

Postpartum hemorrhage (PPH) has been the leading cause of maternal death for at least 30 years, which means that, to reduce global maternal mortality, it is mandatory to reduce deaths due to PPH. 1 2 3 The incidence of PPH varies worldwide, ranging from 5% to 15% of all deliveries, being more frequent in low- and middle-income countries. Moreover, PPH is responsible for severe morbidities, including maternal near-miss. It is expected that, for each case of PPH, between 50 and 100 cases of severe maternal morbidity may occur. 4 5 6 7 8 To decrease PPH mortality, health providers need to identify potential the risk factors and, more importantly, to make an early diagnosis and to establish a prompt and effective treatment.

Early identification of these cases is the basis of the adequate treatment; nevertheless, the current diagnostic criteria may lack the accuracy to identify women at risk of death. The diagnosis of PPH is based on the assessment of the amount of blood loss, usually by visual estimation, which has several limitations, including underestimation of the total volume of the loss. 9 10

In the clinical practice, therapeutic actions depend more on clinical judgment than only on the volume of blood loss. Healthy women may not show signs and symptoms of hemodynamic instability after bleeding 500 mL, while women with clinical or obstetrical morbidities may exhibit signs earlier. 11 12 The aim is to identify women at risk of complications, regardless of any specific amount of blood loss. 13 In addition to the amount of blood loss, vital signs and clinical conditions could be helpful to identify women at risk of complications, as recognized by some clinical guidelines. 14 15

Health providers usually identify other sources of information to compose their clinical judgment on PPH. Some of the vital signs and clinical conditions used in this decision may include the speed of blood flow, heart rate, arterial blood pressure, respiratory rate, dizziness, mental status alteration, among others. 16 However, it is not clear how health providers make the clinical judgment to identify women at risk of a complication due to PPH. Considering this, we aimed to determine how health providers make clinical judgments and recognize cases of PPH.

## Methods

The present is an exploratory and descriptive study using a qualitative approach through a semi-structured interview technique. Using purposive sampling, we selected and invited to participate professionals who worked at the obstetric ward and obstetric labor room at a reference tertiary-level unit for more than 60 cities, covering 5 million people: Hospital da Mulher Prof. Dr. José Aristodemo Pinotti CAISM/Unicamp (Women's Hospital Prof. Dr. José Aristodemo Pinotti CAISM/Unicamp), a hospital of Universidade Estadual de Campinas (UNICAMP, in Portuguese), Brazil. Data were collected from January to March 2018.

The CAISM is a tertiary service for referral of high-complexity cases that performs more than 2 thousand deliveries a year. Training about PPH is carried out once a year with nursing teams and doctors who work at the obstetric ward. Moreover, lectures are offered to all professionals working at that hospital, as well as specific conferences for residents in Obstetrics and Gynecology. The hospital does not have equipment that helps health professionals quantify the amount of blood a woman loses in the postpartum period. The health professionals were categorized as follows: a) nursing team: nurses and nursing technicians (there are no midwives at the hospital); b) medical residents in Gynecology and Obstetrics during the second and third years of residency; c) obstetricians: medical doctors and professors at the School of Medical Sciences of UNICAMP.

We selected the participants from all health professionals who worked with obstetrics at the hospital. The inclusion criteria were: having worked in obstetrics for at least six months; having experienced or witnessed a case of PPH; and accepting to participate. Those who refused to record the interview and who were not available to join after the primary selection were excluded.

The hospital manager provided a list with the names of all health professionals working in obstetrics to identify possible participants. For each participant category, through a random selection process, we identified the order of participants who should be invited to the study. The sample size was achieved in each category when content saturation was reached.

The interviewers invited the participants and explained the objectives of the study; after having their doubts clarified, all participants signed an informed consent form. The face-to-face interview either was performed at that moment, or it was scheduled to take place in a private location or through a telephone call according to the availability of the participants.

For data collection, a pretested semi-structured questionnaire was made available. The questions were related to the experience of diagnosing PPH and the factors that influence its identification. We also included questions about what should be done to improve the identification of PPH. Subjects were identified by numbers (in order to ensure confidentiality and anonymity), and the interviews were recorded and transcribed afterwards.

A thematic analysis of the data was conducted based on Patton. 17 The transcription of the interviews was read, and the seemingly essential words or phrases were highlighted according to the objectives of the study. Based on these important words or phrases, and to group them by similarity, we created codes that were applied to the text. Then, we analyzed thematically each set of texts based on the categories proposed by our objectives.

The present article describes the analysis of the following categories: a) perception of the severity: “there is something wrong with the women”; b) difficulties in the early diagnosis of PPH; and c) the process to improve obstetrical care. The Institutional Review Board at UNICAMP approved the research protocol (under CAAE 71178117.8.0000.5404).

## Results

We interviewed 27 health providers: 9 obstetricians (medical doctors), 7 medical residents, 6 nurses, and 5 nursing technicians. The longest interview lasted 27 minutes, and the shortest, 5.45 minutes. There was no refusal to participate. Table 1 shows the characteristics of the sample.

**Table 1:** Characteristics of the study sample

Gender	n (%)
Female	22 (81.5%)
Male	5 (18.5%)
Age*	
20 to 29 years	7 (27%)
30 to 39 years	10 (38.5%)
40 to 49 years	6 (23%)
≥50 years	3 (11.5%)
**Schooling**	
High school	2 (7.4%)
Higher education	14 (52%)
Graduate studies	11 (40.6%)
**Occupation**	
Obstetrician	9 (33.3%)
Resident	7 (26%)
Nurse	6 (22.2%)
Nursing Technician	5 (18.5%)
**Years of experience**	
≤5	14 (52%)
6-10	5 (18%)
> 10 years	8 (30%)
**Total**	**27**

Note: 1 missing information for the age.

Three main categories of analysis were identified, and they are described and illustrated below.

### Perception of the Severity: ‘There Is something Wrong with the Women’

All participants identified PPH as a life-threatening condition that poses a problem to the health providers.

Although being a concern, there was a lack of knowledge about the frequency and severity of PPH cases, which was justified by the fewer number of severe cases in the institution. In caes of women with risk factors, the staff is more attentive. However, some have assumed that in cases of women without risk factors, providers end up distracted and not paying proper attention to the risk of bleeding during the postpartum period.

The perception that there is something wrong with the women has been reported differently across the four categories of participants. Training differences determine the focus of attention and the actions that each category will perform. The nursing team is more attentive to women's movements and subjective behaviors that differ from normal. It is the way they identify the most serious cases. On the other hand, the medical team is more focused on the objective surveillance of technical parameters, like specific signs and symptoms. After checking the vital signs, symptoms, and performing a clinical examination, when noting that the women are not well, the nursing team notifies the medical team.

*In the daily practice, those who observe the “external” signs are mainly the nursing technicians* . (Obstetrician 14)

In the daily practice, the nursing technicians are the professionals who spend the most time close to the women during the postpartum period; when they realize that something is different from the expected behavior, they call the nurse responsible for the ward. The woman's “complaint” is a piece of critical information for the nursing team, and awareness should be raised whenever a woman does not have complaints.

*Everything that is above the expected evolution has to be watched more closely. Everyone needs to be more suspicious that something may be happening. Every complaint needs to be valued, and the woman who does not complain has to be observed* . (Nurse 4)

On the other hand, some providers assumed that during the process of care, some changes might be neglected and go unnoticed.

*Sometimes, I think it is a bit of a lack of attention. Oh, she is fine, she is talking. Or, sometimes, I don't go to lift her sheet to check the bleeding. If she were bleeding above the normal rate, the multiparameter monitor would be indicating abnormalities...* (Nursing Technician 6)

From the reports of the nursing team, we detected that behavioral changes and the appearance of the woman are very important signs that something is not going well. They say that these signs can be observed even before there are changes in vital signs, or the bleeding is abnormal. They perceived that the women display behavioral changes, such as irritability with the baby, asking to take the baby away, restlessness or paleness, pale, wet, or complaining about weakness, dizziness, and nausea. The nursing team noted that any minor complaint should be considered.

*Irritation/Irritability with the baby, constantly wanting to get out of bed. This behavior is not normal. And what mother does not care about the crying baby who has just been born? These are signs that we monitor also* . (Nurse 2)

Irritability was considered a confounding factor. Women need attention if they become irritated with the baby, feeling uncomfortable in the bed, and wanting to get out of bed frequently. The intervewees consider that, in general, women are happy with their babies and want to be close to them. Nurse and nurse technicians have mentioned that it is possible to differentiate women's mental confusion and disorientation from tiredness due to labor. Other symptoms are also considered very important to identify women at risk of complications, such as when they cannot get up from bed to take a shower, or when, at bath time, their blood pressure drops, and they feel discomfort.

The medical team said they are called by the nursing staff to evaluate the women when there is an issue such as those aforementioned. Doctors are more attached to vital signs and physical examaniations than behavioral signs / symptoms. When noted, doctors mentioned pallor, darkening of vision, lethargy, dizziness, tiredness, and disconnected speech. Medical residents reported, on the other hand, that the main issue for observation and action is the amount of bleeding. Table 2 shows some of the words and strategies used to identify PPH for each category of health professionals.

**Table 2:** Words and criteria used by each category of professionals to identify women at risk of complications due to PPH and the amount of bleeding

Category	Words used to identify the problem	Words used to determine the amount of bleeding
**Nurse technicians**	Irritability with the baby, asking to take the baby away, uneasiness, or the woman turns pale, wet, or complaining about weakness, dizziness, nausea, constantly wanting to get out of bed	Subjective, visual estimate, provider experience
**Nurses**	Every complaint needs to be valued	Subjective, visual estimate of blood loss in the sheets, provider experience
**Medical residents**	The amount of bleeding, vital signs	Subjective, “insight,” visual estimate of blood loss in the sheets and compresses, velocity and intensity of blood loss, vital signs, provider experience
**Obstetricians**	Pallor, darkening of vision, lethargy, dizziness, tiredness, disconnected speech	Subjective, massive number of clots, visual estimate of blood loss in the sheets and compresses, vital signs, provider experience

Among all participants, one thing was unanimous: the perception of the amount of postpartum bleeding is subjective. The health provider is the one who will decide if the amount of blood is normal or abnormal. It is worth noting that there was no available resource to assist the professional in quantifying this bleeding, except their own experience, which can be critical for an early-treatment action.

*We trained our eyes to identify when the bleeding is abnormal. Sometimes, when I have doubts, I call my supervisor for a second opinion* . (Nursing technician 6)

The participants reported that they use some strategies to determine if the amount of blood loss is average. One participant stated they go back to the operation theater to count the number of compresses used during delivery to identify a PPH case. Others evaluate if the bleeding is contained to the bed or if it has spilled to the floor, if there are any clots, and if the bleeding is very intense. Medical residents, for example, realize something is wrong when the vaginal bleeding is highly continuous, running down the bed and with many clots.

In any case, it is important to monitor bleeding even if the woman has delivered without any complications. One participant pointed out that the bleeding should be checked even if the woman is sleeping, because she may be in hypovolemic shock rather than asleep.

### Difficulties for Early PPH Diagnosis

Several participants mentioned some difficulties in the daily practice that work as a barrier to the early identification of PPH cases. The work overload has been related to suboptimal care during the postpartum period. This situation becomes worse when there are few staff members, and it is not possible to check the status of the woman as preconized by the institutional protocol.

*When you have too much work to do, you become negligent. You stop observing, and when you decide to perform a clinical evaluation, the woman has bled profusely without you noticing* . (Obstetrician 16)

It was mentioned that the residents do not have enough experience to perform the early identification of PPH, despite the training offered by the institution. A nursing technician reported that inspection of the uterine tonus sometimes increases women's pain; therefore, they do not use the necessary strength to perform a proper evaluation.

*I remember I did not perform* [the uterine massage] *with the necessary strength. If I did not feel so much pity, maybe I would have have seen the big clots, and we could be started the treatment for PPH before* . (Nursing technician 6)

The distance between the rooms and the nursing station is another difficulty mentioned. Not all women are accompanied at all moments by relatives, who could help identify that there is something wrong.

### The Process to Improve Obstetrical Care

Most health providers interviewed requested a handbook or an easy-to-access flowchart with guidelines on PPH diagnosis and treatment. Due to inexperience, the residents often forget the sequence of steps to treat PPH. Furthermore, besides containing the suggested treatment sequence, the material regarding PPH management should have information about laboratory exams, fluid administration, and blood bank protocols. It has also been recommended that a better criterion to define PPH should be created. Some have cited alternative triggers to start PPH treatment, besides the visual estimate of the blood loss or changes in vital signs.

*A visual scale may be useful. There is the Shock Index. However, we don't have a clear cut-off point to start the treatment. But it is an excellent index, and I think… maybe it should have a more objective diagnostic criterion.* (Obstetrician 25)

Moreover, the medications used to treat PPH should be available at all wards, inside the emergency carts, eliminating the need to go to the pharmacy when necessary. Some interviewees suggested periodic training to improve the diagnosis and treatment of PPH. Otherwise, the training should be performed every time the institution's protocol changes.

*When you perform training, they* [the residents] *become more attentive, but, after a while, they relax their vigilance again. So, the training should be performed a couple of times during the year.* (Obstetrician 26)

The discussion of PPH cases after their resolution was pointed out as a possibility to review and learn from the mistakes and to appreciate potential successful behaviors. In addition, a permanent audit of cases that had a bad maternal outcome should be performed to verify what happened and to implement new actions.

Teamwork and communication should be increased and valorized. Doctors should value more the women's subjective changes reported by the nursing team and clinical evaluation should be performed immediately.

## Discussion

Our findings show that all categories of health providers are very worried about the early identification of PPH to avoid maternal mortality/morbidity. However, the strategies they use to diagnose are different. The nursing team is more alert to behavioral changes as potential early signs/symptoms of PPH. On the other hand, physicians look for objective changes to diagnose and start the treatment. They tend to privilege protocols, changes in vital signs or the estimation of blood loss to diagnose PPH. This difference is probably due to differences in training and the long period that the nursing team spends in close contact with the puerperal woman.

Adequate perception is fundamental, since the nursing team identifies some abnormality and notifies the medical team to initiate the treatment. Subjective and objective assessments complement each other, and should be part of the decision tree to start treatment.

Some authors 18 have proposed using clinical experience and intuitive decision-making as an essential tool for the early recognition of PPH. More experienced health professionals should help less experienced staff to identify any early signs/symptoms of PPH. 18 In the present study, the health provider's experience in assisting women in childbirth and the postpartum period was considered relevant support in the decision-making.

This finding also corroborates the data in the literature, in which intuition/feeling and professional experience are reported to play a critical role in clinical judgment in terms of classifying bleeding according to its severity. 10 19 In the present study, for the diagnosis of PPH, the health professionals consider the amount of blood loss, as well as other individual criteria, like vital signs, but also the provider's experience and intuition/feeling.

Nevertheless, other studies 10 12 20 21 have shown that health providers often underestimate blood loss after childbirth, even after training. The underestimation is even more considerable when the postpartum bleeding is higher. Although there are physical methods to quantify bleeding, their implementation did not decrease morbidity, and it is costly. 9 22 23 24 It is worth noting that there was no available resource to assist the professionals in quantifying this bleeding, except their own experience, which was cited as the most critical factor for early diagnosis.

Diagnosing PPH based on a visual estimate of blood loss is difficult and inaccurate. 10 19 25 26 To enhance the accuracy of PPH diagnosis, some authors 13 18 25 propose to use clinical signs (like the shock index and other clinical components) as an adjuvant tool to perform a visual estimate of blood loss as part of the decision tree to promptly start the treatment for PPH.

In a clinical scenario, simulations and clinical reconstructions have been performed, increasing self-efficacy and reducing stress. However, the long-term benefits of these strategies have not yet been proven. 10 27

Work demand and lack of staff were also pointed out as components challenging the early recognition of PPH. Staff problems, fatigue, and work overload deteriorate the quality of care and potentially decrease the vigilance for a possible PPH diagnosis. 28 29 Facing difficulties and lack of resources, health providers have developed inexpensive strategies that help them identify abnormalities in the postpartum period. These suggestions can have an effect on the diagnosis and prevention of PPH, such as setting PPH protocols in strategic spots and promoting ongoing educational courses. These courses help providers learn to implement a sequence of treatment interventions, and should also include teaching on the importance of non-clinical components such as teamwork, appreciation, communication, and facility readiness.

The present study has demonstrated some gaps, differences, and barriers to the early diagnosis of PPH. Nevertheless, it has offered no solutions. It has already been established that problems with communication are a leading cause of medical errors, including the delay in the diagnosis of PPH. But there is insufficient discussion on the fact that care can be improved with the implementation of bundles of attention, such as the “obstetric hemorrhage bundle”. Such efforts address the reality of different perspectives through education, formal team training (such as shared mental models, conflict resolution, drills, checklists, hemorrhage carts, to name a few elements). This kind of “bundles intervention” may an excellent alternative to implement the necessary changes to improve the early diagnosis and treatment of postpartum hemorrhage. Additional research should assess its feasibility, acceptability, effectiveness and the best strategies to implement it. 30

The present study has some limitations. We included professionals from a university hospital; therefore, we expect that the staff should be more trained than in smaller hospitals. We also included professionals that work in the obstetric ward, and the majority of PPH cases in hospital settings happens in the obstetric room. The present is also a qualitative study, and cannot be generalized; however, it can help scientists to better understand how clinicians develop the clinical judgment for PPH diagnosis. This information can be further investigated using different methodologies to identify factors that could be part of a decision tree for the diagnosis and treatment of PPH.

Caregivers believe teamwork and communication should be improved, as they are considered essential non-clinical components of the diagnosis and management of PPH. Besides the visual estimate of blood loss, the nursing team is attentive to behavioral symptoms like irritability and “constantly wanting to get out of bed” to identify women developing PPH, while the medical staff follow protocols and look for objective signs, such as altered vital signs.

## Conclusion

Besides objective evaluations, the subjective perceptions of health care providers are involved in the clinical judgement regarding the diagnosis of PPH, and this should be included in a broader diagnosis strategy.
